# A multi-view convolutional neural network method combining attention mechanism for diagnosing autism spectrum disorder

**DOI:** 10.1371/journal.pone.0295621

**Published:** 2023-12-08

**Authors:** Mingzhi Wang, Zhiqiang Ma, Yongjie Wang, Jing Liu, Jifeng Guo

**Affiliations:** 1 College of Computer and Control Engineering, Northeast Forestry University, Harbin, China; 2 College of Computer Science and Engineering, Guilin University of Aerospace Technology, Guilin, China; National University of Sciences and Technology, PAKISTAN

## Abstract

Autism Spectrum Disorder (ASD) is a neurodevelopmental condition whose current psychiatric diagnostic process is subjective and behavior-based. In contrast, functional magnetic resonance imaging (fMRI) can objectively measure brain activity and is useful for identifying brain disorders. However, the ASD diagnostic models employed to date have not reached satisfactory levels of accuracy. This study proposes the use of MAACNN, a method that utilizes multi-view convolutional neural networks (CNNs) in conjunction with attention mechanisms for identifying ASD in multi-scale fMRI. The proposed algorithm effectively combines unsupervised and supervised learning. In the initial stage, we employ stacked denoising autoencoders, an unsupervised learning method for feature extraction, which provides different nodes to adapt to multi-scale data. In the subsequent stage, we perform supervised learning by employing multi-view CNNs for classification and obtain the final results. Finally, multi-scale data fusion is achieved by using the attention fusion mechanism. The ABIDE dataset is used to evaluate the model we proposed., and the experimental results show that MAACNN achieves superior performance with 75.12% accuracy and 0.79 AUC on ABIDE-I, and 72.88% accuracy and 0.76 AUC on ABIDE-II. The proposed method significantly contributes to the clinical diagnosis of ASD.

## Introduction

Autism Spectrum Disorder (ASD) is a complex and heterogeneous neurodevelopmental disorder that manifests in early childhood with deficits in social communication, limited repetitive sensorimotor behaviors, and attention problems [1]. ASD typically persists into adulthood and affects about 1% of the global population, with males being four times more susceptible to the condition than females [2]. Elsabbagh et al.’s survey indicated that one in 70 children worldwide has autism. The United States has one of the highest rates of ASD, with an estimated 168 out of every 10,000 children affected in 2018. Currently, clinical interviews and behavioral observation are primarily used to diagnose ASD. The absence of pathological and physiological markers hinders the DSM-based diagnosis of ASD, making it difficult and leading to the potential misdiagnosis of children with the condition, ultimately hindering optimal interventions [3].

The advent of neuroimaging has given rise to functional magnetic resonance imaging (fMRI), a technique used to measure brain activity by calculating changes in local blood oxygenation that correspond with adjacent brain activity. This method enables computer researchers lacking experience in psychiatry or psychology to analyze data obtained from imaging and infer results for individuals with mental disorders [4]. Quantitative analysis of brain imaging data has provided invaluable biomarkers resulting in more precise diagnoses of brain diseases. Machine learning, supported by fMRI, has been extensively utilized by researchers in detecting and identifying ASD, establishing a more objective and scientific approach toward diagnosis. Thus, urgent action is required to develop a neuroimage-based diagnostic method for ASD to reduce delays and misdiagnosis caused by conventional diagnostic processes.

The Autism Brain Imaging Data Exchange (ABIDE) collects data from various international sites. It classifies them into two distinct subsets, ABIDE-I and ABIDE-II, comprising functional and structural brain imaging datasets, respectively. Researchers are increasingly interested in using fMRI data to detect ASD, leading to several studies based on ABIDE datasets. For instance, Iidaka (2015) [5] employed probabilistic neural networks to classify rs-fMRI data of individuals below 20 years of age. Another study by Dvornek et al. (2017) [6] involved training a long short-term memory model on ABIDE data, achieving an accuracy rate of 68.5%. Structural and demographic information has also been incorporated alongside fMRI data to diagnose ASD. Jiang et al. (2020) [7] proposed a graph convolutional network (GCN)-based model that incorporated non-imaging information such as gender and site, achieving an accuracy of 73.1%.

In the field of medical diagnosis, traditional machine learning methods have been applied in the early stages [8, 9]. Abraham et al. (2017) [10] calculated connectivity measures across all regions and employed support vector machines (SVM) to categorize 403 ASD patients and 468 healthy controls, having verified various methods extensively. Another study by Reiter et al. (2021) [11] classified the data of 686 subjects using a random forest algorithm (RF)-based classifier training model, according to gender and the different degrees of functional connectivity (FC). The average accuracy obtained was 67.5%, with the highest accuracy being 73.75%.

In recent years, machine learning methods, including neural networks and deep learning methods [12, 13], have been utilized to diagnose ASD [14–18]. Heinsfeld et al. (2018) [19] categorized 1,035 participants based on their ASD status, where 505 participants had ASD and 530 were healthy controls. The study conducted feature engineering to evaluate the different paired Pearson correlation coefficients. To extract low-dimensional features, the researchers designed two stacked denoising autoencoders (SDA). The weights of the autoencoders were then utilized in the multilayer perceptron (MLP) classifier to classify the participants. The study further tested their models on 17 sites in the ABIDE database and reported an average accuracy of 52%. The researchers attributed the lower accuracy rates to the limited data samples from a particular site that led to underfitting. Another study by Rakhimberdina et al. (2020) [20] proposed a multi-model ensemble method to construct population graphs for diagnosing ASD. The researchers developed multiple population graphs by exploiting different combinations of imaging and phenotypic features and evaluated their performance on the ABIDE dataset. They used a neural network architecture to combine multiple graph-based models together, and achieved an accuracy of 73.13% and an AUC of 0.75. Yao et al. [21], in 2021, improved the graph convolutional neural network to identify brain diseases with an accuracy of 67.3%. Alsaade et al. (2022) [22] evaluated the performance of three deep learning models, VGG19, NASNETMobile, and Xception [23], in classifying ASD on Kaggle’s facial feature dataset. The experimental results reported that Xception achieved the highest accuracy of 91%. It is vital to note that ASD diagnosis lacks reliable biomarkers, and even though certain methods achieve high accuracy, clinical diagnosis is still a challenging task. In recent years, researchers have been dedicated to unraveling the mechanisms of ASD and identifying reliable biomarkers [24]. Wang et al. [25] trained an attention-based graph neural network to automatically diagnose ASD by integrating graph data. The study indicates that the model places more emphasis on brain regions related to the social-brain circuit, default-mode network, and sensory perception network. Additionally, the research detected several genes associated with ASD, contributing to the identification of biomarkers for ASD.

Although machine learning have been developed in the diagnosis of ASD for numerous years, the performance indicators are far from reaching the application requirements of medical diagnosis. Deep learning based on convolutional neural networks has been applied to ASD recognition as well, and has shown to be more effective than traditional machine learning. Currently, there are already some works on multi-atlas [26]. Graa et al. [27] proposed a model called MV-LEAP to solve the class imbalance problem in multi-atlas data. The dataset is balanced by generating synthetic minority class samples using the SMOTE algorithm, and tensor canonical correlation analysis is used to align data from different atlases into a shared low-dimensional subspace. This model enhances the performance of multi-atlas data classification. Gurbuz et al. [28] proposed a method called MGN-Net to normalize and integrate the biological network groups from multi-atlas. This method can retain the unique topological characteristics of the multi-atlas network and build a connection template network by embedding the pairing relationships represented. This method is not only suitable for the diagnosis of ASD but also extends to other brain network diseases. Pan et al. [29] not only used multi-atlas but also used non-imaging data as a supplement. Use AAL and HO two atlases to construct the multi-modal graph representation, and use the snowball graph convolution module to learn multi-atlas information. A channel-sharing convolution module is designed to filter out shared information from multi-modal data through a shared weight matrix. This method achieved better results. Wang et al. [30] used multi-source domain adaptation to convert subjects from different sources into the target domain and then used multi-view sparse representation to combine the image information of gray matter and white matter regions for ASD classification. But, development in ASD identification has been slow in recent years. We find that there are few to combine traditional neural networks and CNN. As a well-known fact, autoencoders have strong feature extraction performance, and the rapid development of CNNs in recent years has made it more and more effective in classification. Based on the above analysis, We propose a convolutional neural network method combined with autoencoders to diagnose ASD, aiming at the problems of optimized model structure, and imperfect single scale information. We propose a new model of ASD identification, which uses stacked denoising autoencoders and the self-designed CNN to train the whole ABIDE datasets. The autoencoder feeds the extracted features to the CNN for classification. This study uses multi-view attention mechanism to fuse multi-scale data, which makes up for the shortcomings of the current research that only uses single scale, and makes full use of the information complementarity between scales effectively improving the performance of ASD recognition.

The structure of this paper is given as follows: The Materials and Methods section introduces the basic information of the employed datasets and the preprocessing of the data. The details of the proposed method and model are presented. In the Results and Analysis section, the experimental settings and results are described and analyzed in detail. In the Discussion section, our proposed method and the latest results obtained by researchers are discussed. Finally, in the Conclusion section, we summarize the study and discuss the direction of future development.

## Materials and methods

### Datasets and data preprocessing

As of now, the most comprehensive open-access dataset available for ASD diagnosis is ABIDE. ABIDE has two major subsets, namely ABIDE-I and ABIDE-II. These subsets come with resting-state functional magnetic resonance imaging data, structural MRI data, and phenotypic data [31]. ABIDE-I is constituted by 1112 datasets obtained from 17 different international sites. The subset consists of 539 ASD subjects and 573 control individuals, with ages ranging from 7 to 64 years [32]. In this study, ABIDE-I is the primary dataset. On the other hand, ABIDE-II is used as a secondary dataset to gauge the models’ dependability.

Preprocessing allows reducing the impact of artifacts and noise on the model and prepares the datasets for FC. The preprocessed connectors project presents different strategies for preprocessing ABIDE-I datasets. The Configurable Pipeline for the Analysis of Connectomes (CPAC), a widely-used open source software pipeline, automatically preprocesses and analyzes rs-fMRI data through slice time correction, motion correction, time filtering, normalization, and registration. The functional images underwent linear transformation to get registered to the anatomical space [33]. This software pipeline exhibited the highest performance in previous studies [34] and as such, it was used to preprocess the datasets in this paper. Because the time series data in the downloaded PCP’s dataset was incomplete, we excluded incomplete data and settled on 419 ASD patients and 530 healthy controls as the research sample. In pre-processing the ABIDE-II dataset, we applied the Data Processing Assistant for Resting-State fMRI (DPARSF) pipeline. In this study, the ABIDE-II dataset was exclusively employed for the validation of model performance and robustness. When applying the leave-one-out cross-validation (LOOCV) approach, each sample requires validation, resulting in potentially high computational costs. Typically, researchers opt for a sample size ranging from 100 to 300 and utilize random selection. This approach aims to ensure a balanced representation across different variables, such as site, gender, and age. Based on the aforementioned criteria, we selected 86 individuals with ASD and 110 healthy controls from 19 sites. It is worth noting that, to avoid the potential influence of non-imaging data, we ensured that the 196 subjects under study were evenly distributed in terms of ages, genders, and sites.

The strength of FC between two regions using fMRI is a widely used heuristic for generating features. FC, which approximates the correlation between different brain regions with several measures, is used to differentiate between subjects with ASD and Healthy Controls (HC). Studies have demonstrated that children with ASD have stronger interregional connectivity within the theta band, which differs from healthy children. The difference in the level of connection strength between the ORBsup area of ASD and HC, relative to other Regions of Interest (ROIs), can be leveraged as an essential factor in ASD diagnosis. FC measures, based on the time-series of rs-fMRI brain imaging data, provide an index of the degree of common brain region activation [35]. The most common measure used to approximate FC is the Pearson correlation coefficient. This measure provides an index that correlates two brain regions, with values ranging from 1 to -1. A value close to 1 indicates a high degree of correlation while a value close to -1 indicates inverse correlation between two time-series. It is a reliable indicator of linearity between two-time series, P and Q, given a length *T*, represented as:

$$\rho_{uv}=\frac{\sum_{t=1}^{T}\left(u_{t}-\bar{u}\right)(v_{t}-\bar{v})}{\sqrt{\sum_{t=1}^{T}{\left(u_{t}-\bar{u}\right)}^{2}}\sqrt{\sum_{t=1}^{T}{\left(v_{t}-\bar{v}\right)}^{2}}}$$ (1)

where $\bar{v}$ and $\bar{u}$ are the average values of time series P and Q, respectively. All pairwise correlations are calculated to obtain the characteristic matrix *M*_*n*×*n*_, where n is the number of region of interest (ROI) of divided atlas. Because of the symmetry of the Pearson correlation, we remove the values of the upper triangle, which repeat the values of the lower triangle, as well as the main diagonal of the matrix, which represents the correlation measure with itself. Finally, only the strict lower triangle part is retained. The final retained matrix is expanded into one-dimensional vectors to retrieve feature vectors, and the number of features obtained can be calculated by

$$S=\frac{n\times (n-1)}{2}$$ (2)

where *n* represents the number of ROIs.

In this study, AAL, CC200, HO, Dosenbach160, and EZ were used for experiments. The AAL atlas is divided into *n* = 116 regions, such that there are 6670 features. The CC200atlas is divided into *n* = 200 regions, such that there are 19900 features. The HO atlas is divided into *n* = 111 regions, amounting to 6105 features. Similarly, the number of features in the Dosenbach160 and EZ atlases can be calculated as 12880 and 6670, respectively.

### Improved feature extraction and classification method

ASD classification tasks have conventionally employed either unsupervised or supervised learning methods. Nevertheless, many studies across other fields have demonstrated that combining both methodologies could leverage their strengths, enhancing the performance of the classification tasks. Autoencoders (AEs) are particularly useful in this regard because they not only help with dimensionality reduction, but also can detect cyclic structures that project images of the same class to edges and corners [36]. An improvement on AE is the Denoising Autoencoder (DAE), which addresses the overfitting problem mainly encountered in AE. By adding noise to the input layer, the DAE is enabled to learn how to eliminate the added noise and isolate clear input data, allowing the encoder to extract the most crucial features and acquire more robust input representations. Consequently, the generalization ability of the DAE is substantially increased over that of the general encoder.

In this paper, we utilize the Stacked Denoising Autoencoder (SDA) to extract low-dimensional features, which are further fed to a CNN that we designed for classification. In generating inputs for the autoencoders, we computed pairwise correlations from the input fMRI using the Pearson correlation function. The encoded data in low-dimensional space is then used in the CNN for further feature extraction and classification. Our methodology is outlined as follows.

**Fig 1:**
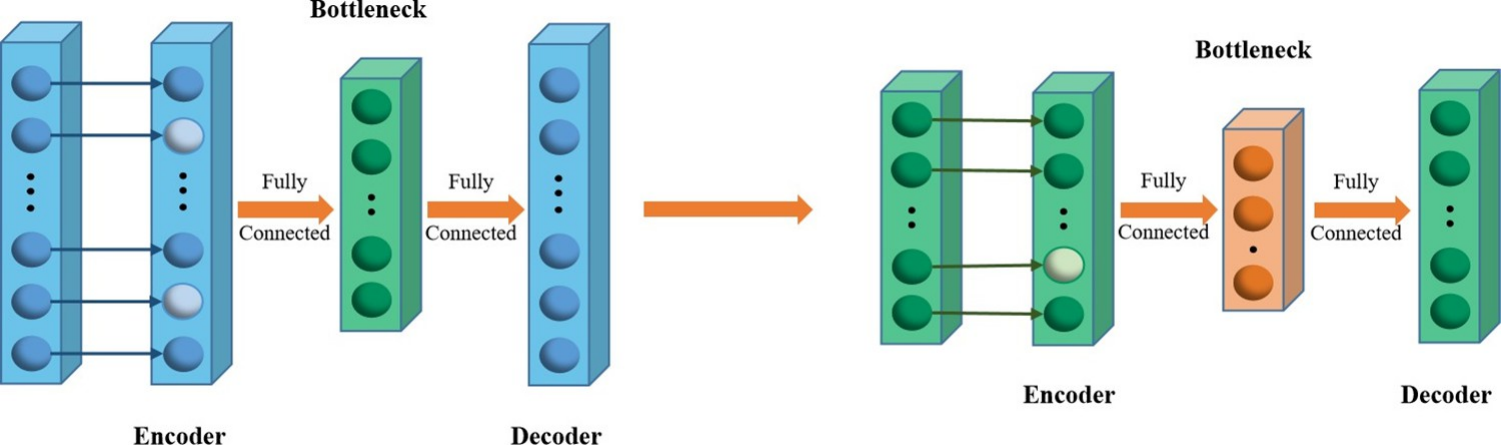
The process of using the stacked denoizing autoencoders for initial feature extraction of raw data.

The architecture of the designed SDA is depicted in Fig 1, consisting of two denoising autoencoders. Each DAE is composed of an encoder, a bottleneck, and a decoder. The encoder receives input data and introduces noise at that layer. The bottleneck layer encodes it into a lower-dimensional representation. The decoder reconstructs the original input from the bottleneck layer.

In the study of Wang et al [37], the authors applied their model to various datasets and the results suggested that the number of hidden layer nodes has a significant impact on the performance of the autoencoder. The traditional methods use AE with a fixed number of nodes, but our model designs a more suitable number of nodes according to the characteristics of multi-scale. We designed the number of nodes in each layer of stacked denoising autoencoder for different atlases (AAL, CC200, HO, Dosenbach160 and EZ), as shown in Table 1. Data damage rates for the first and second DAE were 30% and 10%, respectively. The second DAE has the hidden layer output of 2000 nodes uniformly, which is convenient for the input of the CNN.

**Table 1:** The stacked denoizing autoencoders structure of AAL, CC200, HO, Dosenbach160, and EZ.

	DAE-1 (Data corruption: 30%)			DAE-2 (Data corruption: 10%)		
Atlas	Input layer	Hidden layer	Output layer	Input layer	Hidden layer	Output layer
AAL	6670	3330	6670	3330	2000	3330
CC200	19900	9950	19900	9950	2000	9950
HO	6105	3050	6105	3050	2000	3050
Dosenbach160	12880	6440	12880	6440	2000	6440
EZ	6770	3380	6770	3380	2000	3380

**Fig 2:**
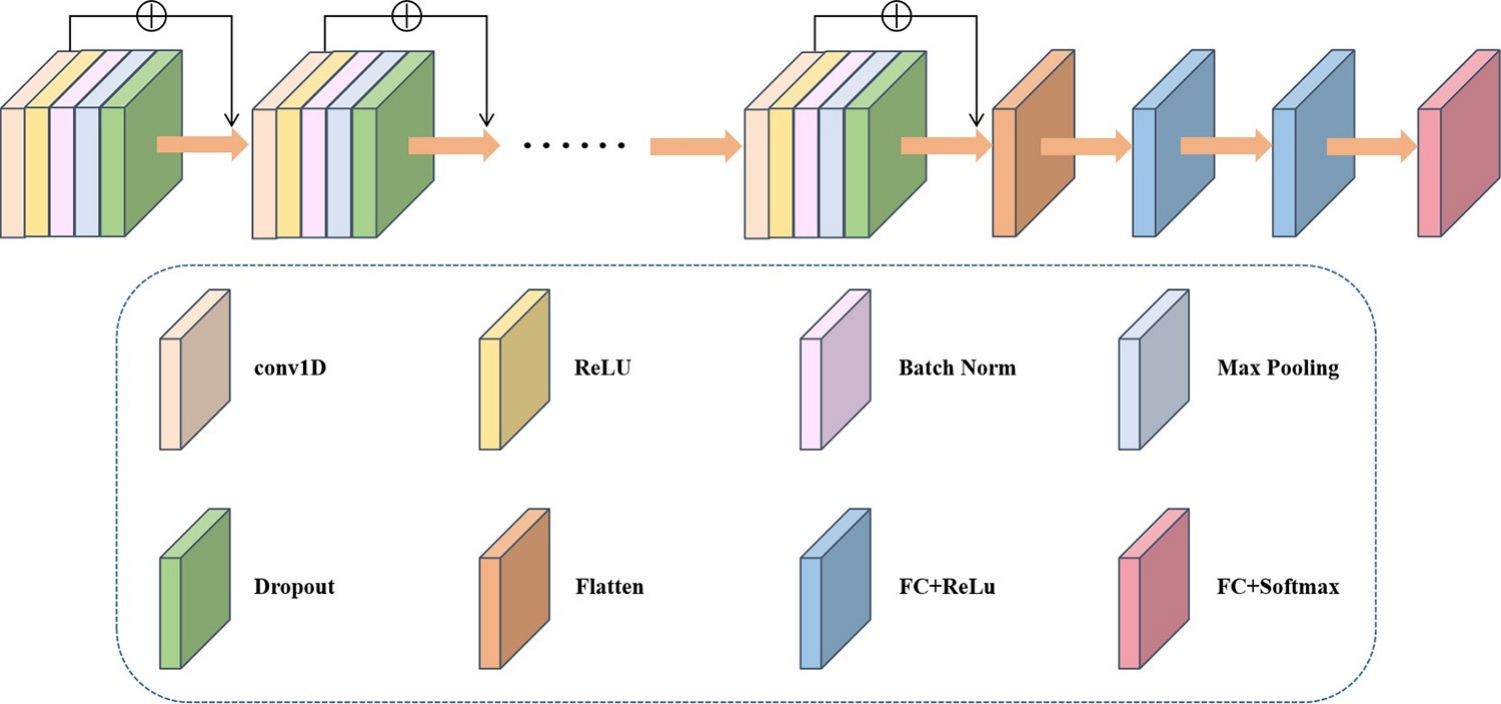
The framework of self-designed convolutional neural network (self-designed CNN).

In our proposed CNN, the 1D convolution layer is immediately followed by the ReLU activation function layer, batch normalization layer, max pooling layer, and dropout layer. After several convolution operations, the one-dimensional data is passed through a Flatten layer before it’s fed into a fully connected layer with a ReLU activation function. In our classification task, we use Softmax as the final activation function for the fully connected layer to obtain the output. Fig 2 provides a clear framework of our proposed CNN for a single scale. Although the depth of the network is limited, overfitting can still be an issue due to the small size of the data samples, particularly with an excessive number of convolutional operations. We address this potential problem by controlling the convolutional operations, adding an L2 regularization term to limit model parameters, and reduce the risk of overfitting. Additionally, we add skip connections between two convolutional layers, in line with the deep residual network, as illustrated in Fig 2.

This approach can enhance feature representation, while also addressing the issues of vanishing and exploding gradients. Additionally, it aids in alleviating information loss resulting from convolutional operations. Max pooling reduces the size of feature maps, leading to a disparity between the sizes of input and output. To tackle this issue, we introduce skip connections, where the input’s feature map is directly added to the output. This is accomplished through the use of identity mapping. In more detail, when the input feature map has dimensions of H×W×C and the convolutions yield a feature map of size H′×W′×C′, if either H≠H′ or W≠W′, skip connections can introduce a 1×1 convolutional operation to adjust the input feature map’s channel count, aligning it with the output channel count. This ensures that the output size of skip connections matches the output size of the convolutional operation, facilitating the connection between two convolutional layers. The operation of skip connections is represented by Formula 3.


Output=Conv(x)+G(x) (3)

In this formula, *x* represents the input feature map, Conv(*x*) represents the feature map obtained by adjusting the input channels using a 1×1 convolutional operation, and *G*(*x*) represents the resulting output feature map obtained through the convolution operation. This process ensures uniform feature map dimensions, facilitating a seamless connection between the two convolutional layers.

### Improved convolutional neural network with multi-view attention mechanism

**Fig 3:**
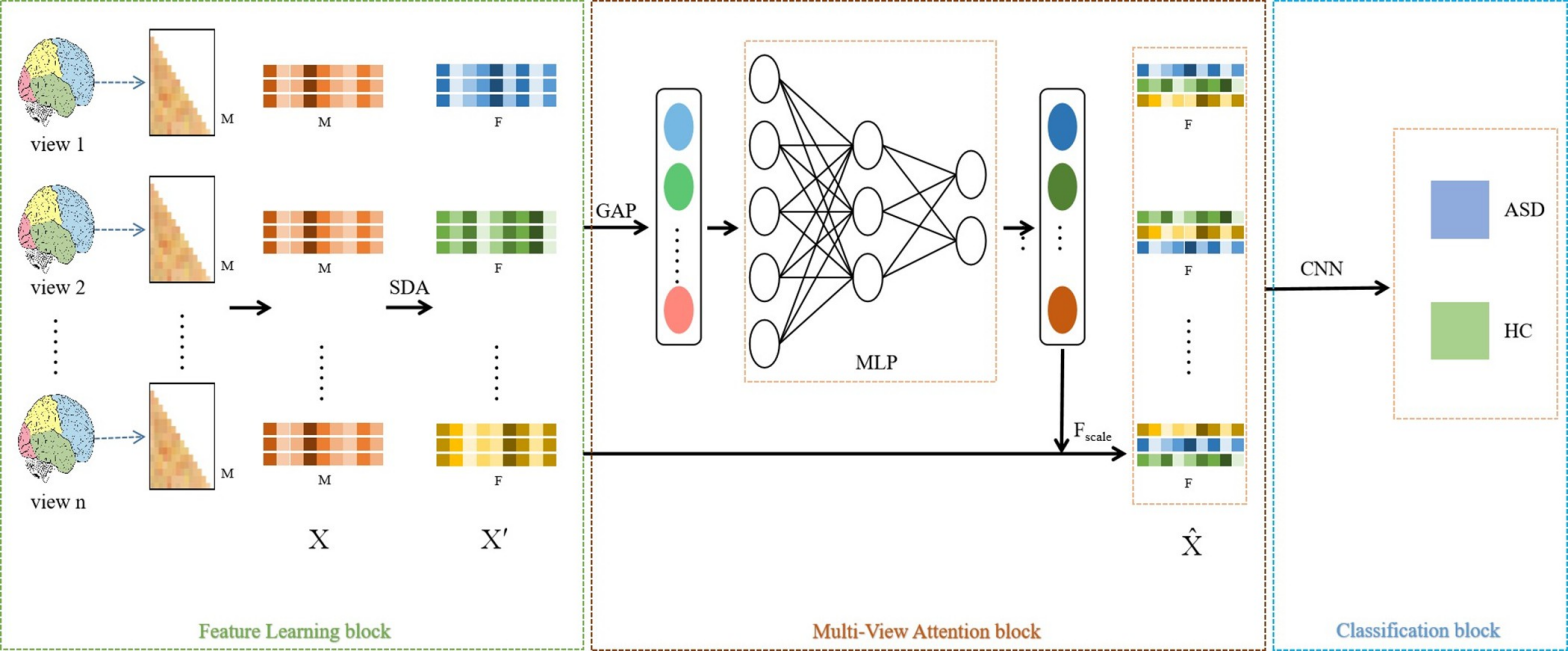
Overall structural framework of our proposed MAACNN.

Most research studies have used only a single atlas as a research object, regardless of whether they employ SVM machine learning methods or CNN deep learning methods, despite the varying information content among different atlases [38]. Our proposed method integrates a multi-view convolutional neural network with a stacked denoising autoencoder, and further incorporates an attention mechanism (MAACNN), to fully consider the complementary nature of multi-scale information. Fig 3 shows that the proposed MAACNN comprises a Feature Learning block, a Multi-View Attention block, and a Classification block.

Within the Feature Learning block, the extracted feature dimensionality may vary depending on the selected view. M, which represents the original feature dimension value, has no fixed value. The raw, preprocessed features are input to the Feature Learning block to generate new representations for all views. The multi-scale raw feature X∈R^N×M^ is represented as X′∈R^n×N×F^ via SDA. N and n respectively denote the number of subjects and views. During Feature Learning, features are standardized and dimensionality is reduced from the non-unique M dimensions to the unique F dimensions.

In other words, for each individual subject X with n views, the original features are mapped from an M-dimensional space to n separate F-dimensional spaces, with each F-dimensional feature containing unique characteristics specific to each view.

The Multi-view Attention block integrates a representation X′ from n F-dimensional spaces and features two modules—the sameness module and the difference module. The sameness module directly inputs X′ to F_scale_.

The second is the attention mechanism distribution learning module. Firstly, Global Average Pooling (GAP) aggregates each view into a single representation by capturing the global features from the feature matrix. This means that the X′∈R^n×N×F^ is transformed into $\tilde{X}\in R^{n\times 1}$. Subsequently, the aggregated $\tilde{X}$ is fed into the MLP to learn the view weight vector $C=\{c_{1},c_{2},\ldots ,c_{n}\}\in R^{n}$. Lastly, the new representation $\hat{X}\in R^{N\times F}$ is gathered by F_scale_ with view weight vector. $\hat{X}$ is generated from X′ as shown in Formula 4.

$$\hat{X}=F_{\mathrm{scale}}(X^{\prime },C)=\sum_{i=1}^{n}c_{i}x^{\prime }{}_{i}$$ (4)

where $\hat{X}$ means the final representation of the multi-view attention block aggregation, and n represents the number of views.

The input to the self-designed CNN in the Classification block, shown in Fig 3, is the new representation embedded into the binary distribution space as $\hat{X}\in R^{N\times F}$. The final classification results are obtained from the Softmax activation function of the CNN’s last layer, as depicted in Fig 2.

## Results and analysis

### Experiment settings

The experiment was conducted on the Linux platform and executed through the Python language. Specifically, the experiment was conducted and debugged on an Ubuntu 18.04 server equipped with an E5-2680 V4 @2.40GHz CPU and a GeForce GTX 3060 GPU. The training process of the proposed model is implemented using the PyTorch framework. Formula 4 includes the adjustable variable n, which can be set to 2, 3, or 4, depending on the number of views in the experiment. Additionally, the F-dimension is fixed at a value of 2000. In this study, a 10-fold cross-validation approach was employed to train and evaluate the model using the ABIDE-I dataset. LOOCV approach was used to test the model on the ABIDE-II dataset. Notably, feature selection was only performed on the training set in each training process and not the entire dataset to counteract the problem of information leakage. This approach helps maintain the credibility of the model’s performance evaluation, which is critical since it is an issue that many researchers might overlook. As a result of this problem, experimental datasets may have high performance, while new datasets, especially for new patients who require clinical application, may not perform as well. Our methodology guarantees the model’s consistent performance on both new and experimental clinical data. The study adopts the stochastic gradient descent algorithm, with the autoencoder’s momentum initialized at 0.9 and the learning rates set at 0.0001. Each training occurs over 200 epochs.

The proposed model’s performance was evaluated by computing the average value of the accuracy (ACC), sensitivity (SEN), and specificity (SPEC) using a 10-fold cross-validation approach. The calculation process involves computing the mean value of the three performance metrics: ACC, SEN, and SPEC. The formula to calculate this is depicted below.


$$\mathrm{ACC}=\frac{\mathrm{TP}+\mathrm{TN}}{\mathrm{TP}+\mathrm{TN}+\mathrm{FP}+\mathrm{FN}}$$ (5)

$$\mathrm{SEN}=\frac{\mathrm{TP}}{\mathrm{TP}+\mathrm{FN}}$$ (6)

$$\mathrm{SPEC}=\frac{\mathrm{TN}}{\mathrm{TN}+\mathrm{FP}}$$ (7)

TP, FP, TN and FN represent the number of ASD subjects correctly classified, ASD subjects incorrectly classified, HC subjects correctly classified, and HC subjects incorrectly classified in the experiment. In addition, we use the area under the ROC curve (AUC) for evaluation.

### Comparison of the impact of different scales on the model

To demonstrate the impact of multi-scale feature representation and attention mechanism, the MAACNN model was employed to perform experiments on five scales (AAL, CC200, Donsebach160, HO, and EZ), and the results of these experiments are outlined in Table 2.

**Table 2:** Performance of single-scale.

Views	ACC (%)	SEN(%)	SPEC(%)	AUC
Dosenbach160	68.72	73.64	58.30	0.72
HO	71.06	75.90	61.65	0.75
AAL	71.69	76.57	62.03	0.75
EZ	70.21	75.04	60.38	0.73
CC200	72.18	77.85	62.41	0.76

The results of Table 2 indicate that the proposed method showed the best performance on the CC200, which was the highest among all five scales. Additionally, the performance indicators of the remaining scales were ranked in the following descending order: AAL, HO, EZ, and Dosenbach160. Except for Dosenbach160, the models achieved an accuracy rate of over 70%, which is already higher than mainstream models. These findings imply that using only a single scale, the proposed feature extraction and classification model produces excellent results.

**Fig 4:**
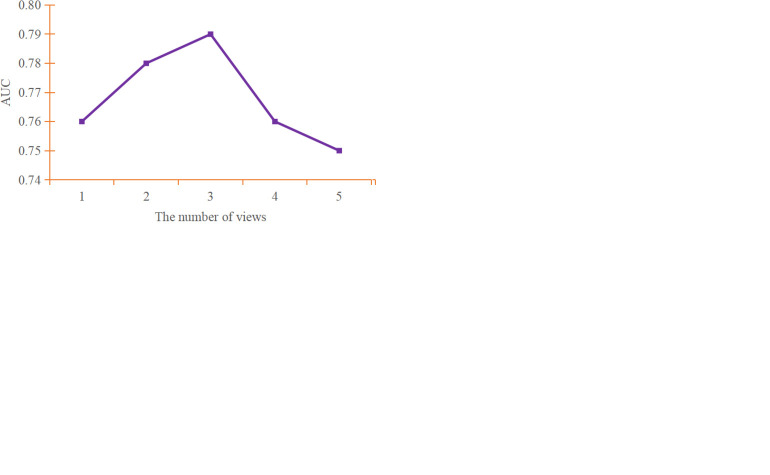
Performance of the model with different number of views. (a) accuracy of the model with different number of views; (b) sensitivity of the model with different number of views; (c) specificity of the model with different number of views; (d) AUC of the model with different number of views.

We utilized multiple views as inputs to the model and computed the final output by utilizing the attention mechanism layer at the end. For our experiments, we performed a 10-fold cross-validation and generated the average performance score of the 10-fold cross-validation trials, as illustrated in Fig 4. The atlases utilized for views in the multi-scale data are displayed in Table 3.

**Table 3:** The views used for each scale.

Number	Views
1	CC200
2	CC200 and AAL
3	CC200, AAL and HO
4	CC200, AAL, HO and EZ
5	CC200, AAL, HO, EZ and Dosenbach160

As depicted in Fig 4, there is a positive correlation between the number of views and the performance metrics of the model, with optimal gains achieved in the combination of three views. Results from two atlases combined into multi-scale exhibited higher model performance than when using any single scale, with the combination of CC200 and AAL achieving an average accuracy of 74.52% and an AUC value of 0.78. When three atlases (CC200, AAL, and HO) were combined into multi-scale, the model achieved optimal results with an accuracy of 75.12%, sensitivity of 80.25%, specificity of 65.71%, and AUC of 0.79. Compared to the best-case scenario of a single scale (CC200), the average accuracy improved by about 2.94%. The performance of the model, however, started decreasing after four views. Taking into account various performance metrics, it was observed that when the number of views reached 5, the model’s performance was even worse than that of a model using a single view. Hence it indicated that simply accumulating views will not result in better outcomes and the views should be adjusted based on the target task attributes.

We propose the incorporation of a multi-view attention mechanism to diagnose autism spectrum disorder, mitigating the impact of atlas division and utilizing complementary information available between different atlases, thereby improving model performance. The experimental results illustrate a considerable enhancement in overall performance by utilizing the multi-scale feature representation, which highlights the superior performance of the proposed multi-view attention mechanism.

### Visualization

**Fig 5:**
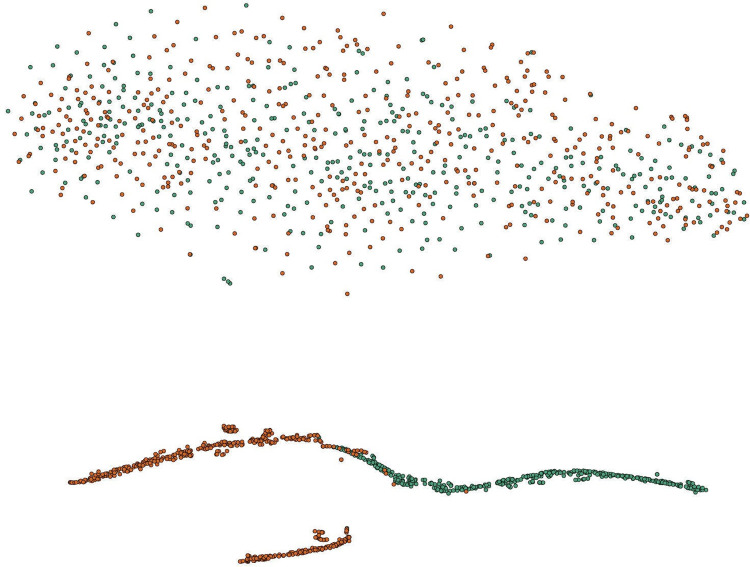
2D feature visualization. (a) t-distributed stochastic neighbor embedding (t-SNE) representation in 2-dimensional space visualized on raw features; (b) feature representation after MAACNN classification. The green nodes represent autism spectrum disorder (ASD) subjects, and the orange nodes represent healthy controls (HC).

The t-SNE technique [39] was utilized for 2D feature visualization to observe the proposed method’s ability in feature fusion and classification. For ease of observation, the distribution of features before model input and after classification under the same data size are presented, and the visualization results are displayed in Fig 5.

The distribution of the raw features can be seen in Fig 5(A), where the nodes of both ASD and HC types are distributed in a disorderly manner and have no obvious boundaries. This also indicates that it is difficult and ineffective to classify directly using the raw features. The node embedding visualization for classification using the proposed method in this paper is shown in Fig 5(B), where nodes of the same type are clustered together and distributed very closely, illustrating excellent performance in sensitivity and specificity. The subgraphs corresponding to HC and ASD nodes have overall similarities. The algorithm clearly distinguishes between ASD patients and typical control group, reflecting the differences between the categories. This indicates that the fused multi-scale features exhibit better intra-class clustering performance and inter-class discriminability, demonstrating the outstanding performance of the method.

## Discussion

Our proposed diagnostic classification method is based on multi-view convolutional neural networks with an attention mechanism and incorporates stacked denoising autoencoders for classifying brain disorders using rs-fMRI data. This study provides the following contributions:

1. The model utilizes two stacked denoising autoencoders to extract features, and a self-designed CNN for classification. By combining supervised and unsupervised learning, it was successful in achieving the desired outcome.
2. The paper introduces a method for diagnosing ASD automatically using multi-scale (multi-atlas) data from subjects. It was demonstrated that appropriate multi-scale data fusion produced better results than single-scale data classification. The proposed model achieved excellent performance.
3. The paper proposes the use of multi-view convolutional neural networks, which utilize the complementary information between different scales. The attention mechanism is used to fuse multi-scale data, achieving high overall performance.

### Performance evaluation

We evaluated the performance of MAACNN by comparing it with baselines and recent studies. HOFC [40], DAE [16], and ASD-DiagNet [41] used a single-scale strategy as the baseline, while recent studies such as GroupINN [42], FC-KNN [43], and MVS-GCN [44] utilized a multi-scale strategy.

GroupINN combines node grouping regularization and non-negative constraints to effectively capture complex relationships between brain regions and identify the most informative subnetworks, thereby improving the performance and interpretability of the model.

FC-KNN is based on rs-fMRI data and addresses the challenges of constructing and analyzing functional connectivity networks, as well as individual variability. It learn pairwise associations between ROIs using a multi-graph fusion method and perform feature fusion and classification using L1 SVM.

MVS-GCN integrates both graph structure and multi-task graph embedding to enhance the classification performance and identify potential functional subnetworks. This method constructs multiple views by generating multiple sparse-level brain network views through multiple thresholding to capture potential correlations between different views, and uses view consistency regularization to ensure the consistency of the model across views. At the same time, prior information of the subnetwork structure is added to the regularization term to enhance the importance of subnetworks in embedding learning, thereby using brain network embedding optimization methods to provide effective ASD diagnosis.

Table 4 reports the experimental results, where the best results are highlighted in bold.

**Table 4:** Performance comparison of various methods.

Category	Method	ACC(%)	SEN(%)	SPEC(%)	AUC
single-scale	HOFC	71.61	61.58	**78.73**	0.77
	DAE	67.31	77.89	54.10	0.64
	ASD-DiagNet	69.56	61.32	67.27	0.67
multi-scale	GroupINN	63.60	61.52	57.36	0.63
	FC-KNN	71.65	65.12	76.35	0.76
	MVS-GCN	69.89	70.18	63.05	0.69
	MAACNN	**75.12**	**80.25**	65.71	**0.79**

Based on the data in Table 4, we emphasize the following observations:

1. The MAACNN model we proposed has significant advantages in accuracy and sensitivity. In particular, the sensitivity reached 80.25%, the only one among all comparative experiments that exceeded 80%, indicating that the detection results accurately captured the presence of the disease. Compared with HOFC and FC-KNN, the AUC of MAACNN was improved by nearly 2% and 3%, respectively. In addition, compared with ASD-DiagNet, which also aims at ASD diagnosis, MAACNN achieved a 5.56% improvement in accuracy. More importantly, compared with the graph-based approach MVS-GCN, although the graph structure learned more phenotype data as a supplement, the complex graph structure had a negative impact on the feature learning of GCN. Even with the best parameters selected, our model achieved a 5.23% improvement in accuracy. These results indicate that our proposed method is effective in ASD diagnosis.
2. Compared to traditional methods, the performance of single-scale strategies is generally inferior to that of multi-scale strategies. The HOFC method achieved the highest result in the single-scale strategy, while MAACNN improved the ACC by 3.51%. In comparison with the baseline method DAE, it can be seen that deep learning methods based on CNNs are significantly superior to traditional methods based on AEs. In our model, we used both AE and CNN simultaneously, combining unsupervised and supervised learning. The results indicate that this combination can enhance the feature representation and classification capabilities of the model.
3. Compared with the state-of-the-art techniques in recent years, our proposed MAACNN shows significant improvement in ASD recognition. Whether in graph-based or non-graph-based methods, our model can better represent and classify features. MAACNN can fully learn the potential associations between different views, and the multi-view attention block has better representation effect on feature fusion. In summary, the results further support our conclusion and demonstrate the effectiveness and robustness of our model.

It is worth noting that this study used all the data of ABIDE-I as the research object, although there are different levels of noise, our model is more realistic than the method using a small sample. It is more in line with the value of clinical diagnosis. According to the experimental results, our proposed MAACNN achieves the highest level at present for all the ABIDE data, which fully demonstrates the superiority of our overall model. In the experiment, we executed 10-fold cross-validation on the whole dataset to test the method proposed in this study. We achieved the highest accuracy compared with the existing methods. The higher AUC shows that stronger model classification capability. In summary, the proposed method has higher model performance and better clinical reference value for the diagnosis of ASD.

We compare the proposed method with other methods under the same brain atlas AAL. Here, we choose HOFC [40], ASD-DiagNet [41], FC-KNN [43] and MVS-GCN [44] methods to conduct experiments using AAL atlas. The experimental results are shown in Table 5.

**Table 5:** Performance of different methods under AAL atlas.

Views	Method	ACC(%)	SEN(%)	SPEC(%)	AUC
AAL	HOFC	71.61	61.58	78.73	0.77
	ASD-DiagNet	67.55	63.40	69.21	0.68
	FC-KNN	71.65	65.12	76.35	0.76
	MVS-GCN	67.14	68.74	62.40	0.67
	MAACNN-AAL	71.69	76.57	62.03	0.75
AAL, CC200 and HO	MAACNN	75.12	80.25	65.71	0.79

As can be seen from Table 5, in the case of using only single view AAL atlas, the comprehensive performance of our model (MAACNN-AAL) is much better than ASD-DiagNet and MVS-GCN, and similar to HOFC and FC-KNN on ACC and AUC, both of which have very excellent performance. There is still much improvement in the specificity of our model. Notably, our proposed model is more prominent in sensitivity, especially since our model is diagnostic for medical images, and high sensitivity indicates that a large fraction of the samples that actually have the disease are correctly recognized as patients (high true positive rate). High sensitivity means that the model is able to capture the actual patients effectively, which is very important for diagnosing the disease. MAACNN-AAL outperforms other single view models with ACC and SEN of 71.69% and 76.57%, respectively. There is a great improvement in model performance in MAACNN using a multi-view model that includes AAL.

**Fig 6:**
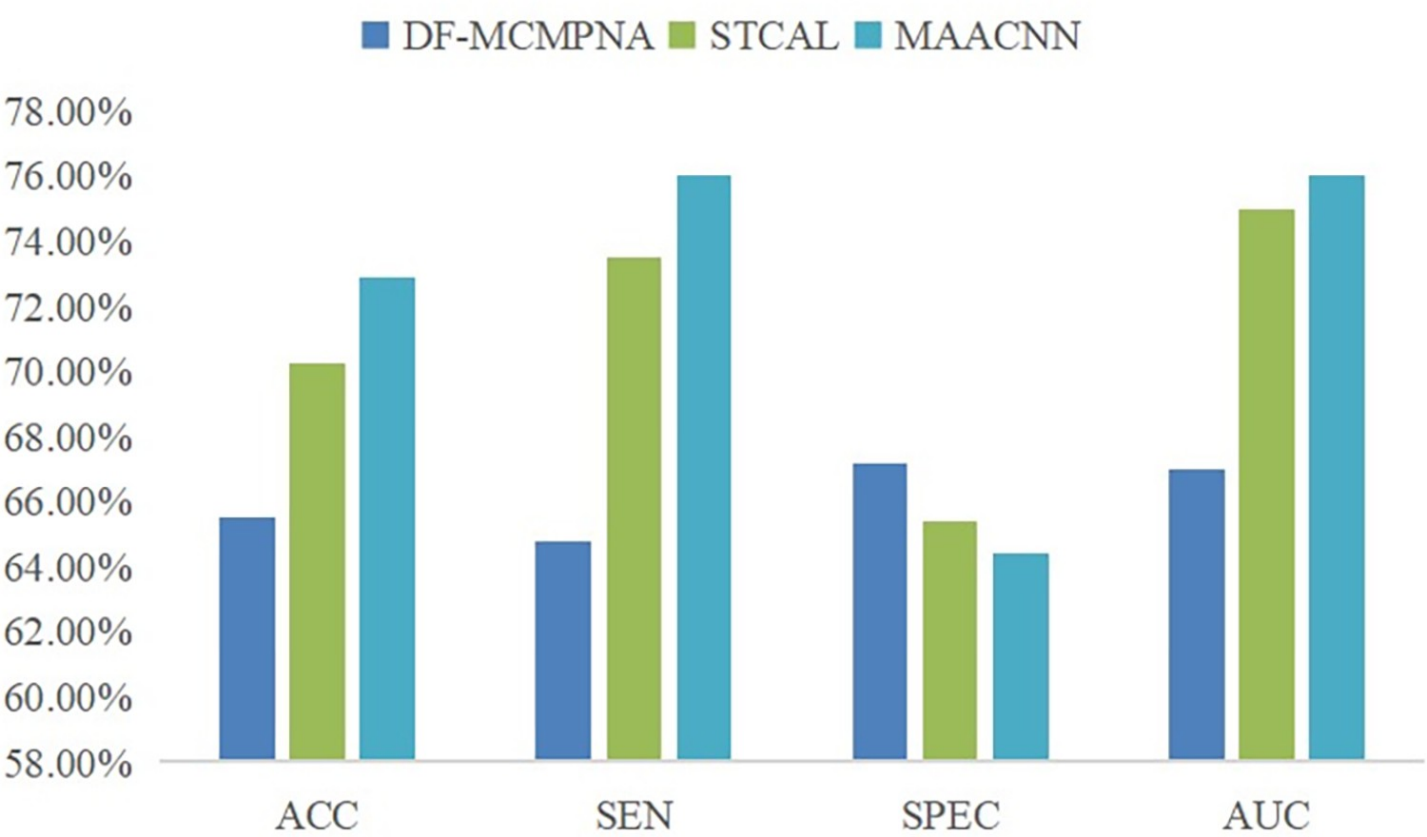
Performance of three methods on ABIDE-II dataset.

To demonstrate the robustness of the MAACNN model, we conducted a validation on the ABIDE-II dataset. We selected the latest research results conducted on ABIDE-II in the past two years as a comparative experiment. Ji and Li [45] proposed a DF-MCMPNA framework to extract and aggregate long-range multi-channel topological features. A multi-channel message passing mechanism and a channel-shared neighborhood aggregation mechanism are used to recursively extract remote multi-channel topological features. The STCAL model by Liu et al. [46] includes a guiding co-attention module to simulate the cross-modal interactions between spatial and temporal signal patterns. Fig 6 shows the LOOCV results of the three methods under the same conditions.

From Fig 6, it can be observed that in terms of accuracy, sensitivity, and AUC, our proposed MAACNN model achieved the best results on the ABIDE-II dataset. The multi-channel message passing and neighborhood aggregation mechanism of the Deep Forest framework can effectively recursively extract remote multi-channel topological features, achieving a LOOCV accuracy of 65.51%. A sliding cluster attention module was introduced to address the global feature dependency of self-attention mechanisms in fMRI time series. Using LOOCV on the ABIDE-II dataset, an AUC of 0.75 was achieved, indicating high overall performance. Our proposed MAACNN not only utilizes a multi-view attention mechanism block to learn the correlations between different views, but also effectively combines unsupervised and supervised learning. Using LOOCV on the ABIDE-II dataset, we achieved an accuracy of 72.88%, sensitivity of 76.02%, specificity of 64.39%, and AUC of 0.76. MAACNN achieved the best performance in both ABIDE-I and ABIDE-II, regardless of whether 10-fold cross-validation or LOOCV was used, demonstrating the effectiveness and robustness of our proposed model and contributing to its application in clinical practice.

### Ablation study

In order to verify the enhancement brought by the proposed module to the model performance, we conducted an ablation study to evaluate the effectiveness of SDA and multi-view attention. We use self-designed CNN as the baseline model for the first set of experiments, and in order to ensure the consistency of the hyperparameters, the traditional recursive feature elimination (RFE) is used directly after calculating the Pearson correlation coefficient to reduce the F-dimensional down to 2000. the second set of experiments adds SDA, thus verifying the validity of SDA. The third set of experiments adds multi-view attention mechanism and does not use SDA. The fourth set of experiments we fuse the proposed modules to evaluate the performance of our proposed MAACNN model. The results of the ablation experiment are shown in Table 6.

**Table 6:** Results of ablation experiments.

Method	ACC(%)	AUC
Self-designed CNN	70.23	0.73
Self-designed CNN + SDA	71.80	0.74
Self-designed CNN + multi-view attention	73.19	0.77
Our proposed model	75.12	0.79

Table 6 demonstrates the impact of different enhancements on CNN’s classification performance through a 10-fold cross-validation approach. In the case of using only CNN, the model achieves ACC of 70.23% and AUC of 0.73. Considering the intricacy of high-dimensional data and the limitations of the original feature representation, we employed SDA for feature extraction and representation. With SDA, the model’s ACC improved to 71.80%, and the AUC increased to 0.74. This suggests that after undergoing feature extraction via our designed SDA, the original features possess enhanced expressive capacity, leading to a significant improvement in model performance.

The introduction of the multi-view attention mechanism enables the comprehensive utilization of complementary information across different views. This is evident even when applied to feature data post RFE, where the model’s accuracy can be elevated from 70.23% to 73.19%. By further integrating the aforementioned modules, our proposed MAACNN model achieves an overall ACC of 75.12% and an AUC of 0.79. The ablation experiments affirm that the proposed SDA and multi-view attention mechanisms significantly contribute to the enhancement of model performance. The amalgamation of these modules yields a substantial improvement in the overall model’s capabilities.

## Conclusion

In this paper, our study proposes a classification method for ASD based on multi-view CNN with an attention mechanism that includes stacked denoising autoencoders, which has demonstrated exemplary performance. Our proposed method incorporates SDA and multi-view CNN for feature extraction and classification, achieving an effective combination of supervised and unsupervised learning. The model output is generated through multi-scale complementary information and an attention mechanism, which lays the foundation for automatic ASD diagnosis. Our study has been confirmed by clinicians. Compared with some current methods, our proposed method achieves the highest levels of accuracy and AUC in identifying ASD. The method offers certain advantages and high performance, which indicates that it has promising applications in the automatic diagnosis of ASD. Additionally, the process can also be extended to the diagnosis of other brain diseases, such as Alzheimer’s disease, and bipolar disorder, etc. In future work, we will try to use graph neural networks combined with information about the population to replace CNN and strive for improved performance.
